# Cadherin-mediated adhesion regulates posterior body formation

**DOI:** 10.1186/1471-213X-7-130

**Published:** 2007-11-28

**Authors:** Michael J Harrington, Elim Hong, Oluwafoyinsa Fasanmi, Rachel Brewster

**Affiliations:** 1 Department of Biological Sciences, University of Maryland Baltimore County, 1000 Hilltop Circle, Baltimore, Maryland 21250, USA; 2 Department of Medicine, University of Maryland School of Medicine, 655 W. Baltimore Street, Baltimore MD 21201, USA

## Abstract

**Background:**

The anterior-posterior axis of the vertebrate embryo undergoes a dramatic elongation during early development. Convergence and extension of the mesoderm, occurring during gastrulation, initiates the narrowing and lengthening of the embryo. However the lengthening of the axis continues during post-gastrula stages in the tailbud region, and is thought to involve convergent extension movements as well as other cell behaviors specific to posterior regions.

**Results:**

We demonstrate here, using a semi-dominant *N-cadherin *allele, that members of the classical cadherin subfamily of cell-cell adhesion molecules are required for tailbud elongation in the zebrafish. *In vivo *imaging of cell behaviors suggests that the extension of posterior axial mesodermal cells is impaired in embryos that carry the semi-dominant *N-cadherin *allele. This defect most likely results from a general loss of cell-cell adhesion in the tailbud region. Consistent with these observations, *N-cadherin *is expressed throughout the tailbud during post-gastrulation stages. In addition, we show that *N-cadherin *interacts synergistically with *vang-like 2*, a member of the non-canonical Wnt signaling/planar cell polarity pathway, to mediate tail morphogenesis.

**Conclusion:**

We provide the first evidence here that *N-cadherin *and other members of the classical cadherin subfamily function in parallel with the planar cell polarity pathway to shape the posterior axis during post-gastrulation stages. These findings further highlight the central role that adhesion molecules play in the cellular rearrangements that drive morphogenesis in vertebrates and identify classical cadherins as major contributors to tail development.

## Background

During early embryogenesis, the shape of the vertebrate embryo changes dramatically, as the embryo narrows along the mediolateral axis and simultaneously lengthens along the anterior-posterior (A-P) axis. These morphological changes are brought about by cellular rearrangements, known as convergent extension (CE) movements, that have been studied extensively in the frog and fish embryo (reviewed in [1-3]). In the fish, CE in the mesoderm involves the convergence of lateral mesodermal cells dorsally by directed migration [4,5]. During mid-gastrulation and continuing into early segmentation, axial mesoderm cells (the precursors of the notochord) undergo mediolateral intercalation to extend the dorsal axis [6,7]. At a cellular level, directed migration is mediated by several types of cell behaviors that occur within distinct dorso-ventral domains [4,5]. Axial cells undergoing intercalation at the dorsal midline become tightly packed and polarized along the mediolateral axis [7]. Following gastrulation, the embryonic axis continues to elongate in the posterior (tailbud) region. Kanki and Ho (1997; [8]) identified four distinct stages of tailbud development: tailbud formation (aggregation of marginal cells to establish the tailbud), tailbud extension (cell movement along the ventral side of the yolk), tailbud protrusion (accumulation of cells in the tailbud, resulting in the formation of an aggregate) and tail eversion (tailbud growth away from the yolk cell). At a cellular level, the mechanisms of posterior body formation in vertebrates involve movements and behaviors that are similar to CE and others that are unique to the posterior body [8-11].

CE movements are mediated, in part, by the non-canonical Wnt signaling pathway, also known as the planar cell polarity (PCP) pathway, which was first identified in *Drosophila*. In vertebrates this branched pathway is activated by the binding of Wnt11/Silberblick (Slb) and Wnt5/Pipetail (Ppt) to their seven-transmembrane-domain receptor Frizzled (Fz; [12]) and transduced through the multifunctional protein Dishevelled (Dsh). Another gene in this pathway, Knypek (Kny, or Glypican 4) is thought to potentiate Wnt signaling [13]. Other transducers of the non-canonical Wnt pathway include: Vang-like 2 (Vangl2) (previously known as Strabismus (Stbm) in the zebrafish), a transmembrane protein [14,15,5]; Prickle (Pkl), an intracellular protein containing three LIM domains and a conserved "PET" domain [16-18]; Rho Kinase 2; and members of the Rho family of small GTPases (Rho A, Rac1 and Cdc42), that regulate different cellular responses such as cytoskeletal rearrangements and cell adhesion [19-22]. c-jun N-terminal kinase (JNK), although not specific to this pathway, is required downstream of small GTPases for CE [23,24]. Consistent with their role in CE, vertebrate embryos in which genes in this pathway are either disrupted via mutation/use of dominant-negative constructs or overexpressed, typically exhibit shortened and broadened A-P axes (reviewed in [25,3]).

There is increasing evidence that the cellular rearrangements that occur during CE also require the regulation of cadherin-based intercellular adhesion, as cells reposition themselves relative to one another [26,27]. The cadherins constitute a large superfamily that comprises classical cadherins, protocadherins and cadherin-like proteins such as Flamingo (Fmi). Knockdown or overexpression of several members of this family have been shown to disrupt CE movements during gastrulation. Zebrafish *flamingo1a and b *(*fmi1a, b*) are expressed in mesodermal cell populations undergoing gastrulation. Morpholino (MO) knockdown of these genes prevents the extension of the entire A-P axis, including the prechordal plate and ventral diencephalic precursors. Fmi1 is thought to function in concert with non-canonical Wnt signaling proteins in these mesodermal populations [28], in agreement with the established role of *Drosophila *Fmi (Starry Night) as a PCP pathway component [29,30]. Wnt11 was recently shown to recruit Frizzled7 (Fz7) and Fmi to the plasma membrane, where the latter is thought to modulate local cell contact persistence in the zebrafish gastrula [31]. Paraxial protocadherin (papc), a member of the protocadherin subfamily expressed in the paraxial mesoderm of the gastrulating embryo, is required for dorsal convergence movements during gastrulation in the *Xenopus *and zebrafish [32-34]. Consistent with this observation, overexpression of *papc *RNA triggers gastrulation movements in *Xenopus *animal cap explants treated with low levels of activin [32]. *Xenopus papc *was shown to signal through the small GTPases RhoA, Rac1 and JNK, that also function downstream of the non-canonical Wnt pathway. Although papc is able to bind to the *Xenopus *Fz7 receptor and shares common downstream components with the non-canonical Wnt signaling pathway, papc and Wnt signaling are not redundant for mesodermal morphogenesis [35,34].

In addition to members of the protocadherin subfamily, classical cadherins, including C-cadherin (C-cad) and E-cadherin (E-cad), are required for mesodermal morphogenesis. In the *Xenopus*, C-cad is essential for gastrulation movements [36]. Moreover, activin-induced axial elongation is accompanied by decreased C-cad-mediated cell-cell adhesion [37,38], indicating that the levels of C-cad need to be tightly controlled for proper mesodermal morphogenesis. Interestingly, papc, functioning downstream of activin and independently from non-canonical Wnt signaling, was shown to downregulate the adhesive activity of C-cad in the paraxial mesoderm [39]. E-cad has also been implicated in gastrulation. Following epiboly, zebrafish mesendodermal progenitors upregulate E-cad and become increasingly motile as they migrate along the overlying epiblast towards the animal pole and contribute to axis elongation. When E-cad function is compromised, mesendodermal progenitors fail to elongate and efficiently migrate along the epiblast [40]. Evidence suggests that Wnt11 controls the levels of E-cad on the cell surface and the general cohesiveness of mesendodermal progenitors by regulating E-cad endocytosis via the GTPase Rab5 [41], highlighting again the dynamic modulation of cell adhesion that accompanies morphogenesis. Finally, it was recently demonstrated that a gradient of adhesive activity established downstream of BMP signaling by a classical cadherin, the identity of which is unknown, drives gastrulation movements in the zebrafish [42]. Several members of the cadherin superfamily thus play a critical role in CE during gastrulation and some appear to interact with the non-canonical Wnt pathway to mediate these movements.

In vertebrates, *N-cadherin (N-cad*, a classical cadherin) expression is restricted to neural tissues, the notochord, somites, cardiac and skeletal muscle [43,44]. Loss of *N-cad *function results in a variety of neural tube defects in vertebrate embryos [44-49]. In contrast to its well-characterized role in the neural tube, there is little evidence that *N-cad *is required for the morphogenetic events that shape the embryonic axis during gastrulation or post-gastrulation stages. In the mouse and zebrafish, only late mesodermal defects associated with the formation of somites [44,50-52] or cardiac muscle [53] have been reported in *N-cad *null embryos. We demonstrate here using a semi-dominant *N-cad *allele that *N-cad*, and possibly other members of the classical cadherin subfamily, are essential for tailbud eversion and to a lesser extent for shaping the mesoderm during gastrulation. Supporting these observations, zebrafish *N-cad *is expressed in axial and paraxial mesoderm during gastrulation and throughout the tailbud. In addition, we show that *N-cad *interacts genetically with *vangl2*, a member of the non-canonical Wnt signaling pathway, to mediate tailbud eversion. We further rule out a role for *N-cad *in regulating apoptosis during the onset of tailbud eversion. Together these data point to a central role for cadherins in mediating the cellular behaviors that drive the elongation of the posterior body.

## Results

### *N-cadherin *mutants display a range of tail phenotypes

**Figure 1:**
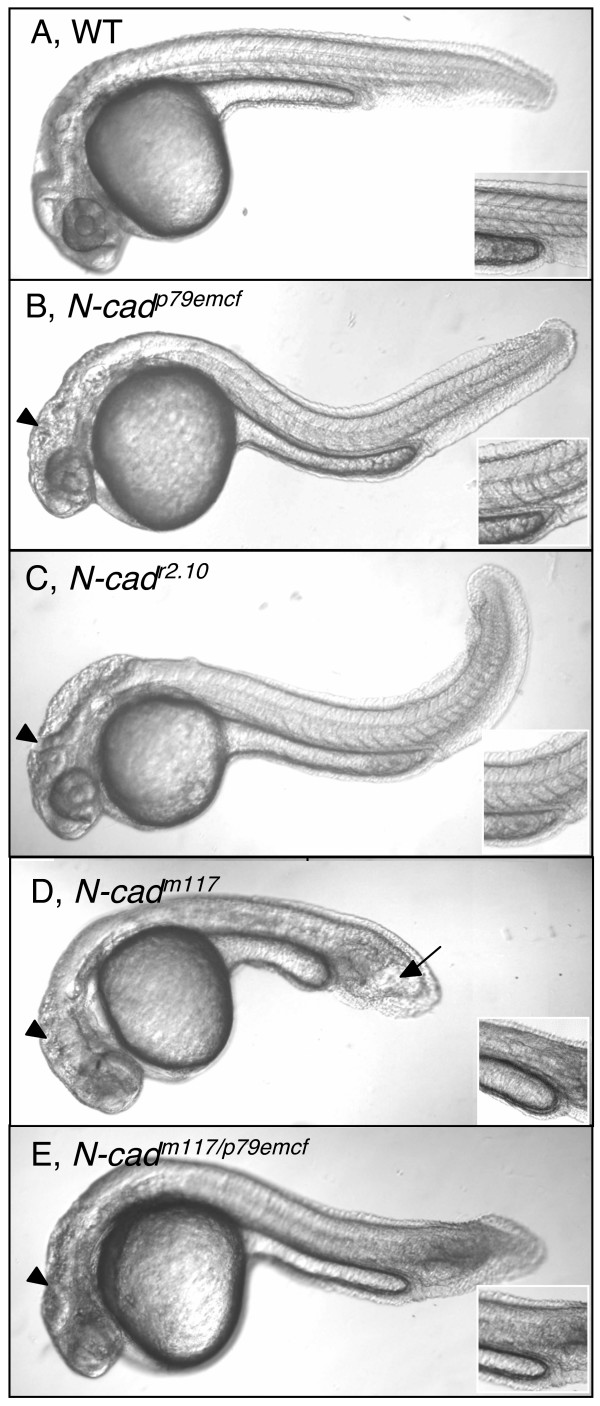
**Loss of N-cadherin function causes axis shortening**. **(A-E) **Lateral views of live 30 hpf zebrafish embryos imaged with Nomarski optics. Anterior is to the left, dorsal is up. Insets show images of somites at the level of the yolk sac extension. Black arrowheads point to brain defects observed in *N-cad *mutants. Black arrow points to the characteristic club-shaped, shortened tail in *N-cad*^*m117 *^homozygous mutants. WT **(A)**, *N-cad*^*p79emcf *^homozygous mutant **(B)**, *N-cad*^*r2.10 *^homozygous mutant **(C)**, N-*cad*^*m117 *^homozygous mutant **(D)**, *N-cad*^*m117/p79emcf *^transheterozygote mutant **(E) **embryos.

Fifteen *N-cad *mutant alleles have so far been generated in forward genetics screens in the zebrafish [54-58], suggesting that this locus is a hot spot for mutation. The most striking phenotype in these mutants is the abnormal brain and eye morphology. However, tail defects with varying degrees of severity have also been reported for several *N-cad *alleles. The tail is curved in *N-cad*^*p79emcf *^homozygous mutants that harbor a missense mutation in the EC5 domain ([58]; Figure 1B). The tail of predicted *N-cad *null mutants that carry truncations upstream of the transmembrane domain (*N-cad*^*fr7*^, *N-cad*^*tm101b*^) or that fail to properly splice the *N-cad *transcript (*N-cad*^*r2.10*^, is either shortened or curved and similar in appearance to *N-cad*^*p79emcf *^([48]; Figure 1C). Surprisingly, the most pronounced posterior phenotype is observed in the *N-cad*^*m117 *^homozygous mutant (previously known as *glass onion*) that contains a substitution of the Tryptophan 2 (Trp2), the second amino acid at the N-terminus of the Cadherin, by Glycine [59]. There is now considerable evidence that classical cadherin-mediated cell adhesion requires a strand exchange process in which Trp2 docks into a hydrophobic pocket in the opposing cadherin and anchors the strand-swap interface [60-64]. The *N-cad*^*m117 *^mutation may therefore block N-cad-mediated cell-cell adhesion by either preventing the strand exchange or, more likely, by preventing the anchoring of the strand-swap interface. *N-cad*^*m117 *^mutants have undefined somitic boundaries by 24 hours post-fertilization (hpf) and a characteristic club-shaped, shortened tail that appears to be filled with vacuoles by 30 hpf ([65]; Figure 1D). Sections through the tail region of these mutants or wild type (WT) embryos injected with *N-cad *MO revealed a "T"-shaped neural tube, previously ascribed to impaired neurulation ([48,49]; Additional file 1 and data not shown). Transheterozygotes for the *N-cad*^*p79emcf *^and *N-cad*^*m117 *^alleles (*N-cad*^*m117/p79emcf*^) exhibit a phenotype intermediate between homozygous embryos for either allele (Figure 1E), indicating that the defects observed in *N-cad*^*m117 *^mutants are caused, at least in part, by misregulation/alteration of *N-cad *function.

### *N-cad*^*m117 *^may block the adhesive activity of other classical cadherins

The posterior defects observed in *N-cad*^*m117 *^mutants suggest that *N-cad *is required for posterior morphogenesis. However, given the more subtle posterior defects observed in presumed *N-cad *null mutants (Figure 1C), it is also possible that *N-cad*^*m117 *^is a gain of function allele, increasing rather than reducing the adhesive activity of N-cad (and possibly of other cadherins). This possibility seems unlikely given the essential nature of the Trp2 residue in mediating adhesion [60-64] and the cell adhesion defects described below. Alternatively, *N-cad*^*m117 *^may alter the activity of other classical cadherins in a dominant or a semi-dominant negative manner. Consistent with this hypothesis, members of this subfamily are known to interact in trans in both a homophilic and a heterophilic manner [66,67]. Moreover, several zebrafish classical cadherins, including N-, E- and M-cadherin, are expressed in the mesoderm during embryogenesis [68,40].

**Figure 2:**
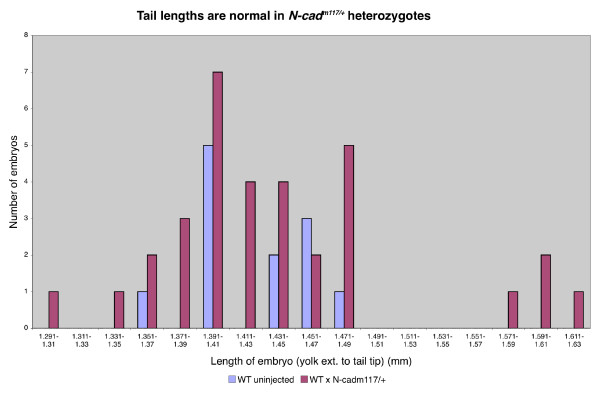
**Length of tail is not altered in *N-cad*^*m117/+ *^heterozygotes**. Measurements of tail length of WT and offspring from a cross between WT and *N-cad*^*m117/+ *^heterozygous embryos at 30 hpf. Tail lengths of *N-cad*^*m117/+ *^heterozygotes were comparable to those in WT embryos.

**Figure 3:**
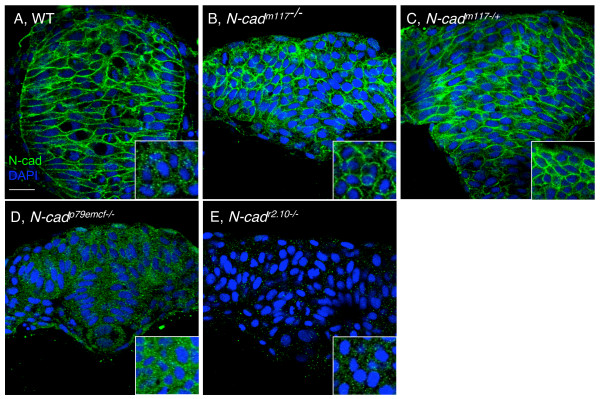
**Expression and localization of N-cad protein in *N-cad *mutants**. Cross-sections through the head region of 7 som stage WT **(A)**, *N-cad*^*m117 *^homozygous mutant **(B)**, *N-cad*^*m117 *^heterozygous mutant **(C)**, *N-cad*^*p79emcf *^homozygous mutant **(D) **and *N-cad*^*r2.10 *^homozygous mutant **(E) **embryos, labeled with α-N-cad (green) and DAPI (blue). Insets show high magnification of cross-sections through the tailbud region of these same embryos. Scale bar, 20 μm.

If *N-cad*^*m117 *^is a dominant or semi-dominant allele, a simple prediction is that *N-cad*^*m*117/+ ^heterozygote embryos should display a phenotype. To address this possibility, WT fish were outcrossed to *N-cad*^*m*117/+ ^heterozygotes and the length of the tail of their offspring, half of which should be *N-cad*^*m*117/+^, was measured at 30 hpf. These measurements did not reveal a bimodal distribution (Figure 2), but rather, a full overlap with measurements taken from a cross between WT fish, indicating that the presence of this allele in a heterozygous configuration may not be sufficient to cause a posterior phenotype. To further explore the possibility that the *N-cad*^*m117 *^is dominant or semi-dominant, we scored the offspring from a cross between two heterozygotes for the presence of neural convergence defects at 7 somites (som), as it has been previously reported that neurulation is impaired in *N-cad *mutants [48,49]. At 6–7 som, *N-cad*^*m117 *^homozygous mutants are readily identifiable, based on the neural tube defects and the lack of somitic boundaries [59]. Cross sections through the head region of these embryos revealed a keel-shaped neural tube (Figure 3B), indicative of impaired neural convergence, whereas WT embryos at this stage have a neural rod (Figure 3A). Interestingly, two out of three "non-mutant siblings" from this cross, had neural convergence defects (Figure 3C), in contrast to the third embryo that had a rod-shaped neural tube (data not shown). Such defects have not been observed in *N-cad*^*p79emcf *^or *N-cad*^*r2.10 *^heterozygotes (data not shown). The simplest interpretation of these observations is that *N-cad*^*m117/+*^heterozygotes have mild neurulation defects. Thus, the *N-cad*^*m117 *^allele appears to be semi-dominant, at least with regards to neural convergence.

To further investigate the reason underlying the difference in severity of the posterior defects in *N-cad *mutants, we also examined N-cad protein expression and localization in several *N-cad *mutants. Interestingly, N-cad protein was mislocalized away from the plasma membrane in *N-cad*^*p79emcf *^mutants (Figure 3D), that harbor a point mutation in the EC5 domain [58]. This finding was confirmed using an antibody against β-catenin, a protein that associates with the cytoplasmic domain of N-cad (data not shown). Consistent with previously published observations, no N-cad protein was detected in *N-cad*^*r2.10 *^homozygous mutants (Figure 3E; [48]). In contrast, normal or slightly elevated levels of N-cad protein were observed in *N-cad*^*m117 *^mutants (Figure 3B) and in putative *N-cad*^*m117 *^heterozygotes (Figure 3C). Together these observations suggest that *N-cad*^*p79emcf*^may function as a null allele, explaining why the phenotype of *N-cad*^*p79emcf *^mutants is very similar to that of *N-cad*^*r2.10 *^mutants. Furthermore, the presence of N-cad protein in *N-cad*^*m117 *^heterozygotes and homozygotes is consistent with this allele being semi-dominant and altering the adhesive activity of other cadherins by heterophilic interactions.

In summary, loss of *N-cad *and other classical cadherin function impairs posterior mesoderm morphogenesis and somite boundary formation, in addition to neural convergence. Moreover, the fact that severe brain but only mild posterior defects are observed in embryos carrying *N-cad *null alleles (Figure 1B, C) suggests that brain development is mostly dependent on *N-cad *function whereas tail developmental may require several cadherin family members with overlapping functions. Since *N-cad*^*m117*^*/N-cad*^*p79emcf *^transheterozygotes have an intermediate phenotype, as compared to their homozygous mutant counterparts, this indicates that *N-cad *does participate in posterior morphogenesis. We focus the rest of this study on the posterior defects.

### Loss of *cadherin *function blocks tail elongation

**Figure 4:**
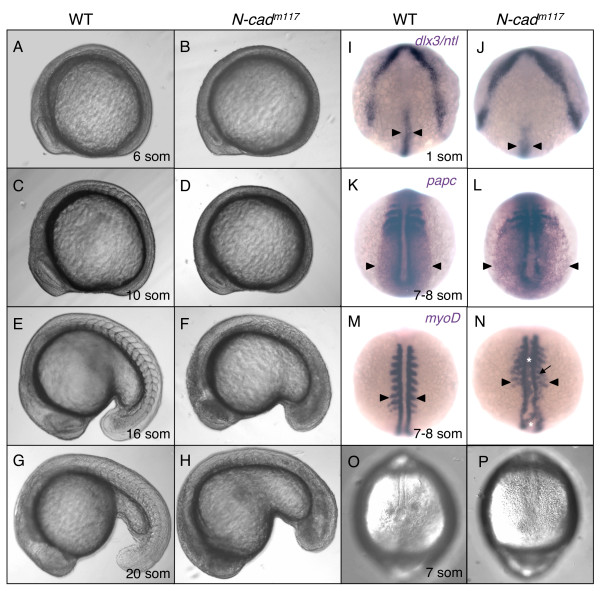
**Mesodermal morphogenesis defects in *N-cad*^*m117 *^mutants**. **(A-H) **Lateral views of WT **(A,C,E,G) **and *N-cad*^*m117 *^mutant **(B,D,F,H) **embryos imaged with Nomarski optics at 6 som **(A,B)**, 10 som **(C,D)**, 16 som **(E,F) **and 20 som **(G,H)**. Anterior is to the left, dorsal is up. **(I-N) **Dorsal view of WT and *N-cad*^*m117 *^mutant embryos at 1 som **(I,J) **and 7–8 som **(K-N) **processed by *in situ *hybridization. **(I,J) **Dorsal anterior view of *dlx3 *and *ntl *expression. Black arrowheads indicate width of NC. **(K,L) **Dorsal posterior view of *papc *expression. Black arrowhead point to the lateral edge of the paraxial mesoderm. **(M,N) **Dorsal view of *myoD *expression. Black arrowheads indicate length of somites. White asterisks indicate ectopic labeling in the axial mesoderm. Black arrow points to ectopic intersomitic *myoD *labeling. **(O-P) **Dorsal posterior view of WT **(O) **and *N-cad*^*m117 *^homozygous mutant **(P) **at 7 som, imaged with Nomarski optics. Abbreviations: som, somite.

The tail defects observed at 30 hpf in *N-cad *loss of function embryos may be indicative of a role for *N-cad *and other cadherins in shaping the mesoderm during gastrulation or in driving the cell behaviors that mediate tailbud growth during late somitogenesis. In order to address these possibilities, *N-cad*^*m117 *^mutants were examined at earlier stages of development for morphological defects typically associated with impaired gastrulation or/and tailbud elongation. Morphologically,*N-cad*^*m117 *^mutants are first identifiable at 2–3 som, based on their neural keel phenotype [48]. However, at this stage of development, the length of the A-P axis appeared normal, even though somitic boundaries were undefined (data not shown). Embryos examined at later stages, ranging from 6 to 14 som, also looked normal with respect to the length of their A-P axis (Figure 4B, D and data not shown). However, axial defects became apparent by 16 som (Figure 4F) and were more pronounced by 18 som and continuing into later stages (Figure 4H). These observations are consistent with a requirement for *N-cad *in tailbud eversion, a late stage in tailbud growth, when the tailbud moves away from the yolk cell [8].

In order to determine whether loss of cadherin function may cause subtle defects in CE movements during gastrulation that were not detectable using morphological criteria, embryos were further analyzed using molecular markers for ectodermal and mesodermal tissues. The extent of axial mesoderm CE was examined at the 1 som stage, using a *no tail *riboprobe to label the axial mesoderm. *N-cad*^*m117 *^mutant embryos were readily identifiable at this stage of development by co-labeling with *dlx3*, a marker for the edge of the neural plate. Indeed, a quarter of the embryos from a cross between *N-cad*^*m117 *^heterozygous parents revealed an enlarged neural plate (compare Figures 4I, J), caused by impaired CE in the neural ectoderm. This phenotype is consistent with the expression of *N-cad *in the neural plate and the known role of *N-cad *in neural convergence [48,49]. The width of the notochord in *N-cad*^*m117 *^mutant embryos was comparable to that of WT siblings (Figure 4J). To test for paraxial mesoderm convergence defects, 7 som stage embryos were examined using *papc *and *myoD*, markers for presomitic paraxial mesoderm and somites, respectively. Presomitic mesoderm appeared laterally expanded in *N-cad*^*m117 *^homozygous mutants (Figure 4L) but not in *N-cad*^*m117 *^heterozygotes (Additional file 2). These results suggest that loss of cadherin function can cause mild mesodermal convergence defects and confirm that the *N-cad*^*m117*^allele *is *not fully dominant. Consistent with the enlarged *papc *domain, somites were slightly wider in *N-cad*^*m117 *^mutants than in WT siblings (Figure 4N). These phenotypes are similar to, although less pronounced than, those observed in non-canonical Wnt signaling mutants, such as *kny *and *trilobite (tri; *which disrupts *vangl2)*. In addition to the lengthening of the somites, *myoD*-positive cells were observed in ectopic positions in *N-cad*^*m117 *^mutants (Figure 4N), suggesting a loss of cell-cell adhesion. This mixing of mesodermal populations was previously observed in embryos in which *papc *is disrupted [69]. Dorsal views of 7 som *N-cad*^*m117 *^mutants imaged using Nomarski optics also revealed the presence of scattered, disorganized cells adjacent to a poorly defined notochord and throughout the tailbud, confirming a loss of tissue integrity in the paraxial mesoderm of *N-cad*^*m117 *^mutants (Figure 4P).

Together, these observations suggest that the cell intercalation behaviors that drive the narrowing and elongation of the dorsal axis (notochord) during gastrulation are normal in absence/reduced function of *N-cad *and other cadherin family members, but the convergence movements in paraxial mesoderm may be slightly defective. In addition, the tailbud fails to undergo proper eversion in *N-cad*^*m117 *^mutants, which most likely accounts for the severe posterior shortening observed in older embryos. This defect may be caused by impaired intercellular adhesion. Given that neural and mesodermal markers are expressed in a regionally appropriate manner in these embryos ([48]; this study and data not shown), cell fate specification is unlikely to be a contributing cause to the posterior defect.

### *N-cadherin *is expressed in the mesoderm during gastrulation and post-gastrulation stages

**Figure 5:**
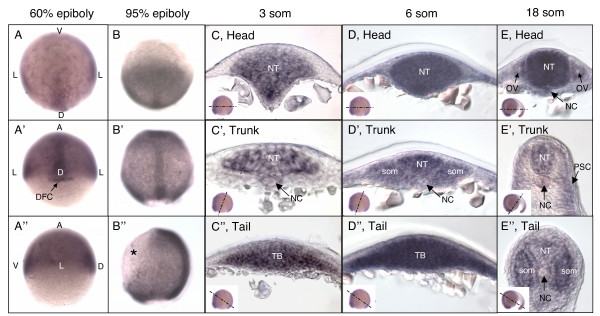
**Expression of *N-cadherin *during gastrulation and somitogenesis**. *N-cadherin *mRNA expression in WT, 60% epiboly **(A-A")**, 95% epiboly **(B-B")**, 3 som **(C-C")**, 6 som **(D-D")**, and 18 som **(E-E") **embryos. Animal views **(A,B)**, dorsal views **(A',B')**, and sagital views **(A",B")**. Asterisk indicates lack of *N-cad *expression in the ventral epidermis. **(C-E") **Cross-sections through the head **(C,D,E)**, trunk **(C',D',E')**, and tail **(C",D",E") **regions. Insets indicate the angle at which the embryo was sectioned. Abbreviations: V, ventral; L, lateral; D, dorsal; A, animal; NT, neural tube; NC, notochord; som, somite; TB, tailbud; OV, otic vessicle; PSC, postmigratory slow cells.

To determine whether the expression pattern of *N-cad *is consistent with a role in mesodermal morphogenesis, *in situ *hybridization was carried out on embryos at different stages of development. A previous report indicated that *N-cad *is not expressed maternally [48], therefore *N-cad *expression was analyzed beginning at gastrula stages. At the shield stage (60% epiboly), *N-cad *RNA is fairly ubiquitous, with higher levels in the dorsal region of the embryo (Figure 5A–A"). The dorsal forerunner cells (DFCs) express particularly high levels of *N-cad *at this stage (Figure 5A'). As gastrulation proceeds (95% epiboly), expression is enhanced in prospective neural tissue and axial mesoderm (Figure 5B–B") and turned off in the ventral (non-neural) ectoderm (Figure 5B").

By mid-somitogenesis, *N-cad *is present ubiquitously in neural tissue with highest levels in the anterior neural rod (Figure 5C, D). Expression in the presomitic (Figure 5C') and paraxial mesoderm (Figure 5D') is prominent in the trunk region. In the tailbud, *N-cad *expression is observed throughout the prospective neural tissue and mesoderm but is conspicuously absent from the epidermis and enveloping layer (Figure 5C", D"). By late somitogenesis (18 som), during tailbud protrusion, *N-cad *expression is very high in anterior neural tissue (Figure 5E), while trunk and tail neural tissue have lower levels (Figure 5E', E"). Trunk somites express *N-cad *in postmigratory slow cells (PSCs) while the tail somites express *N-cad *throughout, as previously described by Cortes et al., 2003[68]  (Figure 5E', E").

### Increased apoptosis does not contribute to the onset of posterior defects

**Figure 6:**
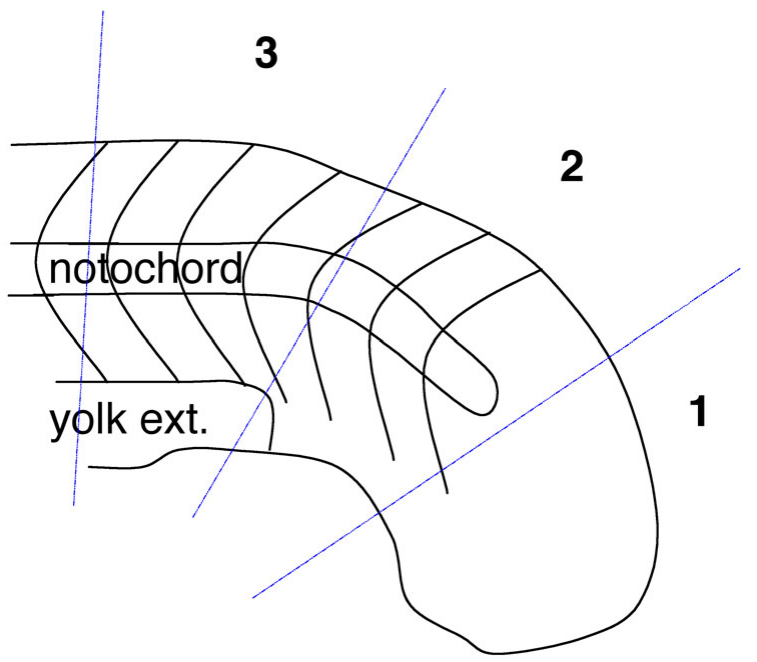
**Diagram of tail regions analyzed for apoptotic cells**. The tails of 18–19 som and 22–23 som embryos were divided into three regions (1, 2, and 3) and analyzed for TUNEL-positive cells. Each region spans approximately 100 μm in length.

The expression of *N-cad *throughout the tailbud is consistent with a role for *N-cad *in mediating the cell behaviors that contribute to tail eversion. However, it is also possible that increased cell death accounts for the posterior defects in *N-cad*^*m117 *^mutants. To investigate this possibility, apoptosis in the tailbud was scored by counting the number of TUNEL-positive cells in the tail region (Table 1 and Figure 6). Staining was performed at the 18–19 som stage (when tailbud eversion defects first become apparent), at the 22–23 som stage and at 30 hpf. At 18–19 som, a slight increase in apoptosis was observed in *N-cad*^*m117 *^mutants compared to WT siblings, however the increase was too low to account for the severity of the tail defects at this stage. Moreover, the number of apoptotic cells at 22–23 som was similar between *N-cad*^*m117 *^mutants and WT embryos. In contrast, a dramatic increase in cell death was observed at 30 hpf in *N-cad*^*m117 *^mutants relative to WT (data not shown). These results indicate that increased apoptosis is unlikely to contribute to the tailbud eversion defect at early stages (19–23 som) but may contribute significantly in shortening the posterior axis of older embryos (30 hpf).

**Table 1:** Apoptosis during tailbud eversion. Number of cells labeled using the TUNEL assay in 18–19 som (A) and 22–23 som (B) embryos is shown as mean ± SD. (1, 2, 3) Tail regions in which the TUNEL-positive cells were counted (refer to figure 6 for illustration). 0.4 ng of *vangl2 *MO was injected into each embryo where specified

**A**	Genotype		18–19 somites		
			
			1	2	3
WT		(n = 4)	2.75 ± 1.26	2.75 ± 2.22	1.75 ± 2.22
*N-cad*^*m117*^		(n = 3)	11.00 ± 3.46	10.33 ± 2.31	7.33 ± 1.53
WT w/*vangl2 *MO		(n = 3)	6.67 ± 5.51	9.00 ± 6.93	10.67 ± 10.07
*N-cad*^*p79emcf*^		(n = 3)	6.33 ± 0.58	13.33 ± 1.15	1.33 ± 1.53
*N-cad*^*p79emcf *^w/*vangl2 *MO		(n = 2)	14.00 ± 8.48	6.50 ± 3.54	3.00 ± 2.83

**B**	Genotype		22–23 somites		
			
			1	2	3

WT		(n = 4)	3.00 ± 3.56	2.00 ± 2.83	3.25 ± 2.36
*N-cad*^*m117*^		(n = 7)	3.14 ± 1.77	5.57 ± 3.21	0.29 ± 0.49
WT w/*vangl2 *MO		(n = 7)	1.29 ± 2.56	0.71 ± 1.25	1.00 ± 1.00
*N-cad*^*p79emcf*^		(n = 8)	2.13 ± 2.03	1.63 ± 1.60	0.50 ± 0.53
*N-cad*^*p79emcf *^w/*vangl2 *MO		(n = 7)	1.86 ± 1.57	0.86 ± 0.69	1.71 ± 1.60

### Impaired movement of posterior axial mesodermal cells underlies the tailbud elongation defects

Zebrafish *N-cad *is known to drive the convergence of neural cells [48,49] and other zebrafish classical cadherins have been implicated in mesodermal morphogenesis ([68,40,70,71,42]; reviewed in [27]). It is therefore likely that *N-cad *and other members of the cadherin family also participate in the cell movements implicated in posterior body elongation. The tailbud is derived from two cell populations, posterior tailbud cells (originating from the ventral blastoderm margin and fated to become tail paraxial mesoderm) and anterior tailbud cells (derived from the dorsal region of the embryo and fated to become axial mesoderm [8]. Cell tracing experiments were therefore carried out to determine whether cell movements of posterior or anterior tailbud cells are impaired in *N-cad*^*m117 *^mutants.

**Figure 7:**
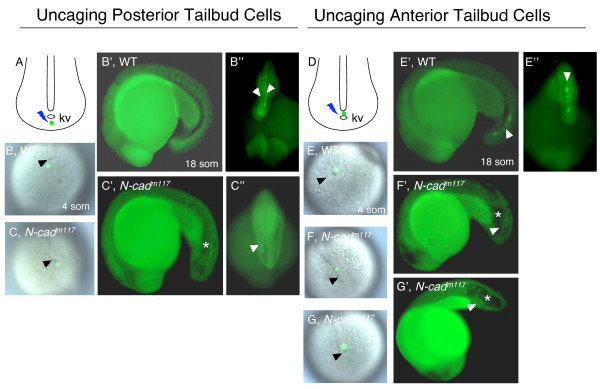
**Impaired movement of anterior tailbud cells in *N-cad*^*m117 *^mutants**. Posterior **(A-C") **and anterior **(D-G') **cells in the tailbud were uncaged at 4 som **(A-C,D-G) **and imaged at 18 som **(B'-C",E'-G')**. **(A,D) **Schematic diagrams of a dorsal view of the tailbud at 4 som, illustrating where the uncaging was done (green dot). **(B,C,E-G) **Dorsal views of the tailbud at 4 som, showing where the uncaging was done (green label), in WT **(B,E) **and *N-cad*^*m117 *^mutants **(C,F,G)**. Lateral **(B',E') **and dorsal **(B",E") **views of 18som WT embryos indicating the position of uncaged cells (white arrowheads). Lateral **(C',F',G') **and dorsal **(C") **views of *N-cad*^*m117 *^mutants indicating position of labeled cells (white arrowheads). Abbreviations and symbols: som, somite; kv, Kupffer's vesicle; asterisk indicates the position of the vacuole in the tailbud, black arrowheads show KV.

Posterior tailbud cells undergo subduction, movement underneath the anterior tailbud cells, at the boundary between the ventral-derived posterior and dorsal-derived anterior cells. Following subduction, posterior tailbud cells move anteriorly and laterally (laterad divergence) to avoid the midline [8]. In order to test whether the movement of posterior tailbud cells was impaired in *N-cad*^*m117 *^mutants, cells immediately posterior to Kupffer's vesicle (KV) were labeled at the 3 to 4 som stage (Figure 7A–C) and the position of their progeny scored at the 18 som stage (Figure 7B'–C"). Labeling in WT siblings revealed either single or double rows of cells adjacent to the dorsal midline, indicating that cells underwent laterad divergence and adopted a paraxial mesoderm fate, as previously reported (Figure 7B'–B", n = 4 out of 4 embryos). Labeled cells in *N-cad*^*m117 *^mutants also appeared to move anteriorly (n = 7 out of 8 embryos), avoiding the midline (n = 5 out of 8 embryos). However, unlike WT cells, they did not organize into rows but rather, were scattered medio-laterally (Figure 7C'–C"). These findings suggest that cadherin-mediated adhesion may not be required for cell behaviors of the posterior tailbud, but are necessary to maintain the general cohesiveness of this tissue.

Anterior tailbud cells are thought to extend into the tail by cell rearrangement and intercalation (a continuation of gastrulation movements, as described in the chick and the mouse, [72,73]) and by enlargement of notochord cells due to vacuolation. To investigate whether posterior extension of the axial mesoderm is defective, cells immediately anterior to KV were labeled at 4 som (Figure 7D–G) and the position of their progeny scored at the 18 som stage (Figure 7E'–G'). Labeling of WT siblings revealed that cells aligned along the posterior midline and adopted a cuboidal morphology characteristic of notochord cells (Figure 7E'–E", n = 2 out of 2 embryos). In contrast, labeled cells in *N-cad*^*m117 *^mutants were observed in ectopic positions, which mostly coincided with the location of large vacuoles in the tail tip. Cells were found associated with the dorsal region of the tailbud (Figure 7F', n = 3 out of 7 embryos), adjacent to the yolk extension (Figure 7G', n = 3 out 7 embryos) or within the vacuoles (data not shown, n = 1 out of 7 embryos). In addition, labeled cells failed to adopt the characteristic cuboidal morphology (n = 7 out of 7 embryos). Thus, cadherins appear to be necessary for the proper positioning of axial mesoderm cells. It is likely that loss of tissue integrity in the tailbud region, as evidenced by the scattering of cells (Figure 4N, P and Figure 7C"), may be the underlying reason for this defect. Indeed, cell-cell adhesion is known to be essential for cell intercalation movements such as those that drive notochord elongation (reviewed in [27]).

*In situ *hybridization on wholemount 30 hpf *N-cad*^*m117 *^mutants using *myoD *(a marker for ventral-derived cells that differentiate into somatic mesoderm) and *no tail *(a marker for anterior-derived axial mesoderm cells that differentiate into notochord) confirmed the uncaging data. *myoD *was expressed throughout the posterior region (although labeled cells did not organize into somites, data not shown), indicating that ventral-derived cells were able to populate the posterior region. In contrast, *no tail *expression was not observed in the tail tip (data not shown), supporting the finding that axial mesoderm does not extend posteriorly in *N-cad*^*m117 *^mutants.

### *N-cadherin *and *vang-like 2 *interact to regulate posterior body morphogenesis

**Figure 8:**
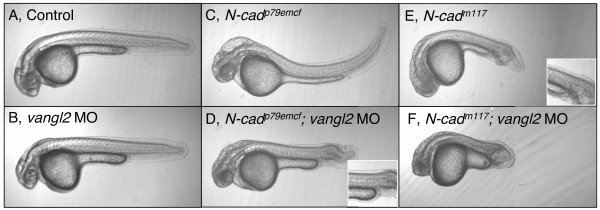
**Genetic interaction between *N-cadherin *and *vang-like 2***. Lateral views of 30 hpf live embryos imaged using Nomarski optics. Anterior is to the left, dorsal is up. Control uninjected (WT) **(A)**, WT injected with *vangl2 *MO (0.8 ng) **(B)**, *N-cad*^*p79emcf *^homozygous mutant **(C)**, *N-cad*^*p79emcf *^mutant injected with *vangl2 *MO (0.8 ng) (*N-cad*^*p79emcf*^; *vangl2 *MO) **(D)**, *N-cad*^*m117 *^homozygous mutant **(E)**, *N-cad*^*m117 *^mutant injected with *vangl2 *MO (0.8 ng) (*N-cad*^*m117*^; *vangl2 *MO) **(F) **embryos. Insets show images of somites at the level of the yolk sac extension.

**Figure 9:**
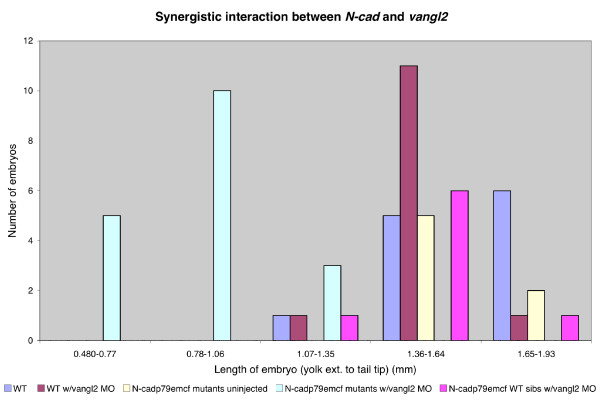
***vangl2 *MO injected into *N-cad*^*p79emcf *^mutant embryos causes tail elongation defects**. Tail lengths of *N-cad*^*p79emcf *^mutant embryos uninjected and injected with 0.8 ng of *vangl2 *MO were measured at 30 hpf. Injection of *vangl2 *MO into *N-cad*^*p79emcf *^mutants had significant effects on the overall length of the embryonic axis.

*N-cad*^*m117 *^mutants have a phenotype that is similar although not identical to non-canonical Wnt signaling mutants such as *tri, kny *and *ppt*, which exhibit shortened A-P axes by early segmentation stages [11]. This raises the possibility that classical cadherins and non-canonical Wnt signaling components have a partially redundant function. A potential interaction between *N-cad *and *vangl2 *was investigated by combining *N-cad*^*p79emcf*^, an *N-cad *allele that displays a very mild tail phenotype, (Figure 1B and 8C), with a low dosage of *vangl2 *MO. Titration of *vangl2 *MO was undertaken to identify a MO concentration at which CE movements were near normal in injected embryos, when assayed at 30 hpf (Figure 8B, Additional file 3). The optimal amount was found to be 0.8 ng, as this is the lowest dosage at which an interaction with *N-cad*^*p79emcf *^was observed. Since injections were done when mutant embryos could not be identified, genotyping was retroactively carried out at 30 hpf. Interestingly, *N-cad*^*p79emcf *^homozygous mutants injected with the predefined low dosage of *vangl2 *MO (*N-cadp*^*p79emcf*^*; vangl2 *MO) had dramatically shortened tails (Figure 8D, observed in 83% of the injected mutants, n = 15 out of 18, Figure 9) relative to controls (Figure 8A), WT injected with *vangl2 *(Figure 8B), uninjected *N-cad*^*p79emcf *^mutants (Figure 8C), and *N-cad*^*p79emcf *^WT siblings injected with *vangl2 *MO (image not shown). The general morphology of *N-cad*^*p79emcf*^; *vangl2 *MO embryos appeared superficially similar to that of *N-cad*^*m117 *^mutants. However, upon closer examination, the phenotypes were not identical as somites formed properly in the trunk region of *N-cad*^*p79emcf*^; *vangl2 *MO embryos and their tails had less vacuoles than *N-cad*^*m117 *^mutants (compare Figures 8D, E).

**Figure 10:**
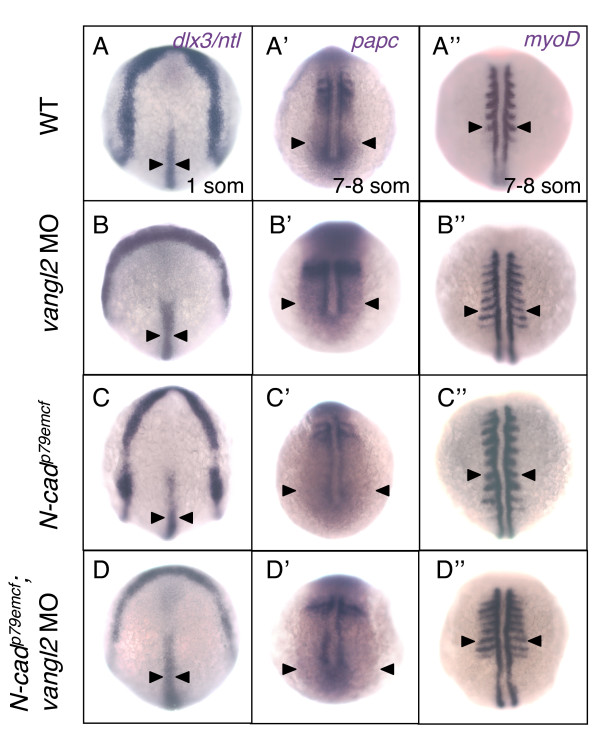
***N-cad*^*p79emcf*^; *vangl2 *MO embryos do not exhibit early gastrulation defects**. Dorsal views of WT **(A-A")**, *vangl2 *MO-injected **(B-B")**, *N-cad*^*p79emcf *^homozygous mutant **(C-C")**, and *N-cad*^*p79emcf*^; *vangl2 *MO **(D-D") **embryos labeled with *dlx3 *and *ntl *at 1 som **(A-D)**, *papc *at 7 som **(A'-D')**, and *myoD *at 7 som **(A"-D")**. Black arrowheads indicate the width of the NC **(A-D)**, the lateral edge of the paraxial mesoderm **(A'-D')**, and the length of the somites **(A"-D")**. Abbreviations: som, somite; NC, notochord.

To address how early the *N-cad*^*p79emcf*^; *vangl2 *MO tail phenotype becomes apparent, embryos were examined at 10 and 20 som. While no axial elongation defects were present at 10 som (tail extension stage), *N-cad*^*p79emcf*^; *vangl2 *MO embryos had severely shortened tails by 20 som (tailbud eversion stage, data not shown). Consistent with a late onset phenotype, *N-cad*^*p79emcf*^; *vangl2 *MO embryos labeled at 1 som with *dlx3 *and *ntl *(Figure 10D) did not exhibit a wider neural plate or notochord, relative to WT injected with *vangl2 *MO (Figure 10B). *papc *labeling of 7 som *N-cad*^*p79emcf*^; *vangl2 *MO embryos revealed a slightly expanded paraxial mesoderm domain (Figure 10D') relative to controls (Figure 10A'–C'), however this phenotype was not more severe than the combined defects observed in WT embryos injected with *vangl2 *MO (Figure 10B') and *N-cad*^*p79emcf *^mutants (Figure 10C'). Likewise, *myoD *labeling at 7 som failed to detect a significant lengthening of the somites in *N-cad*^*p79emcf*^; *vangl2 *MO embryos (Figure 10D") relative to WT embryos injected with *vangl2 *MO (Figure 10B"). It is also noteworthy that *N-cad*^*p79emcf*^; *vangl2 *MO embryos do not exhibit ectopic *myoD*-positive cells, in contrast to *N-cad*^*m117 *^mutants (compare Figures 4N with 10D").

Together these observations suggest that *N-cad *and *vangl2 *interact genetically and that this interaction is primarily required, during post-gastrula stages, for tailbud eversion. Increased apoptosis in posterior regions was ruled out as a likely contributing factor to tail shortening, as the number of apoptotic cells at 18–19 som and 22–23 somites in *N-cad*^*p79emcf*^; *vangl2 *MO embryos was roughly equivalent to that observed in controls (WT, WT injected with *vangl2 *and *N-cad*^*p79emcf*^; Table 1). These findings suggest that impaired cell movements cause the posterior defects. Moreover, the fact that the phenotypes observed in *N-cad*^*m117 *^mutants and *N-cad*^*p79emcf*^; *vangl2 *MO embryos are qualitatively different suggests that *N-cad *and *vangl2 *may not interact to regulate cell-cell adhesion.

### *N-cadherin *and *vang-like 2 *are likely to function in parallel pathways

The genetic interaction between *N-cad *and *vangl2 *is a priori consistent with these genes functioning either in linear or parallel pathways. To distinguish between these two possibilities, the ability of the *vangl2 *MO to worsen the *N-cad*^*m117 *^null phenotype was tested. If these genes function in a linear pathway exclusively, then *vangl2 *MO should have little or no effect on *N-cad*^*m117 *^mutants. *vangl2 *MO was injected at the same low concentration used in combination with the *N-cad*^*p79emcf *^allele (0.8 ng). 100% (n = 8 out of 8 embryos) of the *N-cad*^*m117 *^mutants injected with this dosage of *vangl2 *MO had enhanced tail defects at 30 hpf (Figure 8F) relative to uninjected *N-cad*^*m117 *^mutants (Figure 8E), suggesting that *N-cad *and *vangl2 *function synergistically and in parallel pathways to regulate CE.

It has recently been reported that *Wnt11 *controls the general cohesiveness of mesendodermal progenitors, by regulating E-cad endocytosis [41], indicating that, in some cases, classical cadherins function downstream of the non-canonical Wnt pathway. To further address whether the relation between *vangl2 *and *N-cad *is strictly non-linear, N-cad levels and localization were analyzed in two planar cell polarity mutants, *tri *and *kny*. Cross sections through the tail region of 18 som embryos did not reveal any overt differences in N-cad labeling between WT and mutants (Additional file 4). These findings are consistent with N-cad and the planar cell polarity pathway functioning synergistically and in parallel to regulate posterior morphogenesis.

## Discussion

### Role of *N-cadherin *in posterior mesoderm and neural tube morphogenesis

Several pieces of evidence point to a primary role of *N-cad *and possibly other cadherins in regulating posterior mesoderm morphogenesis. Firstly, the length of the A-P axis in *N-cad*^*m117 *^mutants appears normal until 16 som, which coincides with the onset of tailbud eversion [8]. In contrast, embryos in which members of the non-canonical Wnt signaling pathway such as *vangl2 *and *kny *have been disrupted, show a dramatic shortening of the axis beginning at mid-somitogenesis stages [13,15,5,11]. The early onset of A-P defects in these mutants reflects the role of *vangl2 *and *kny *in mesodermal morphogenesis during gastrulation. Secondly, molecular markers only show a slight impairment in paraxial mesodermal convergence during gastrulation in *N-cad*^*m117 *^mutants. Thirdly, *in vivo *labeling of targeted cell populations in the tailbud revealed that while the anteriorward movement of ventral-derived cells (the precursors of paraxial mesoderm) appears normal, dorsal-derived cells (notochord precursors) are unable to extend posteriorly. This finding is surprising given the widespread expression of *N-cad *throughout the tailbud region. However, several lines of evidence indicate that cadherins are essential for cell intercalation behaviors underlying notochord extension (reviewed in [27]), but may not be required for directed migration of cells, a process that is thought to mostly rely on cell-extracellular matrix interactions mediated by integrins. The extension of the notochord along the yolk ball via cell intercalation is thought to be a contributing force for posterior tail extension [8] and may therefore explain, at least in part, the tail defects observed in *N-cad*^*m117 *^mutants.

The data presented in this paper has focused on N-cad's role in the mesoderm, as CE in this germ layer is thought to account for most of the elongation of the A-P axis. However, N-cad is also required for the morphogenesis of the neural tube (NT). Fate mapping studies have shown that the posterior spinal cord derives from dorsal cells in the epiblast of the anterior tailbud. These precursor cells are thought to become distributed along the entire length of the developing tail by CE-like movements and cell proliferation [8]. The NT in posterior regions of *N-cad*^*m117 *^mutants and WT embryos injected with high levels of *N-cad *MO is not only shortened but also has a characteristic "T" shape, indicative of impaired NT closure [48,49]. Thus, in addition to shaping the mesoderm, *N-cad *is also required for proper posterior NT morphogenesis, consistent with its broad expression throughout the tailbud.

### Why is the *N-cad*^*m117 *^phenotype stronger than that of presumed null mutants?

Several *N-cad* null alleles have been previously described in the zebrafish [48]. Embryos homozygous for these null alleles exhibit mild posterior defects. We have further characterized another potential null allele, *N-cad*^*p79emcf*^, that carries a point mutation in the EC5 domain [58]. Immunofluorescence studies revealed that the *N-cad *protein is mislocalized away from the plasma membrane in these mutants. This may be potentially explained by a misfolding of the protein, such that it is not able to properly insert into the membrane. Accordingly, the phenotype of *N-cad*^*p79emcf *^homozygous mutants is very similar to that of confirmed *N-cad *null mutants. It is possible that the tail curvature defect observed in these embryos may be caused by impaired neurulation but normal mesodermal CE. With stronger *N-cad *alleles (*N-cad*^*m117*^), impaired mesodermal morphogenesis may alleviate this curvature defect while causing a shortened axis.

*N-cad*^*m117 *^mutants have a severe posterior defect that is not observed in *N-cad *null mutants. We propose that this allele is semi-dominant, as mild neural convergence defects were observed in heterozygous siblings. Since *N-cad *is known to interact heterophilically with other members of the classical cadherin family [66,67], *N-cad*^*m117 *^may affect the adhesive activity of these proteins. Alternatively, *N-cad*^*m117 *^may impair the function of other *N-cad *paralogues, recently identified following sequencing of the zebrafish genome.

### *N-cadherin *may function as an adhesion or a signaling molecule

At a molecular level, how could N-cad function to promote cellular rearrangement? As a member of the classical cadherin subfamily, N-cad is known to mediate cell-cell adhesion [43,48,74,75]. Morphogenetic movements such as those that occur during CE require a dynamic regulation of adhesion, as contacts between cells have to be constantly broken and re-established in order for cells to exchange neighbors and locally reposition themselves [26]. In this context, the adhesive activity of C-cad is known to play a critical role during *Xenopus *gastrulation [37,38]. It was recently shown that C-cad's adhesive activity is regulated by *papc*, functioning downstream of activin and independently from non-canonical Wnt signaling [39]. This raises the intriguing possibility that a similar relationship exists between N-cad and Papc in the paraxial mesoderm and may be required for proper mesodermal morphogenesis. There is also strong evidence that classical cadherins can function as signaling molecules (reviewed in [76]). For example, axonal outgrowth in retinal ganglion cells is dependent on the interaction between N-cad and the FGF receptor (FGFR) [77-80]. The invasive activity of N-cad during cancer metastasis also results from a functional interaction with FGFR at the cell surface [81,82]. Other signaling molecules through which cadherins can function to stimulate cell motility is the Rho family of small GTPases, the steady state activation of which increases in the presence of N-cad [83] and Retinal cadherin (R-cad)-mediated cell-cell contact [84]. Activation of these GTPases correlates with increased cell motility [84-86].

Thus, experimental data strongly supports a role for N-cad in both adhesion and signaling. Further elucidation of the role of N-cad and other cadherins in promoting posterior morphogenesis will require assays to distinguish between these two functions.

### Interaction between N-cadherin and the non-canonical Wnt signaling pathway

A genetic interaction between *N-cad *and *vangl2 *(a non-canonical Wnt signaling component) was demonstrated by slightly lowering the levels of *vangl2 *in embryos carrying the *N-cad*^*p79emcf *^allele. *N-cad*^*p79emcf*^; *vangl2 *MO embryos exhibited a dramatically shortened tail, similar to that observed in *N-cad*^*m117 *^mutants. *N-cad *and *vangl2 *were interpreted to function synergistically and in parallel pathways, as lowering the levels of *vangl2 *in *N-cad*^*m117 *^mutants worsened the tail defect in these embryos even further and N-cad levels and localization were not perturbed in *tri *and *kny *mutants. These findings suggest that there are other pathways regulating the distribution of N-cad protein in cells undergoing movement. Moreover, the similar yet distinct phenotype of *N-cad*^*m117 *^mutants and *N-cad*^*p79emcf*^; *vangl2 *MO embryos suggests that N-cad and Vangl2 may not interact to regulate intercellular adhesion but rather some other cell behavior.

There is increasing evidence that regulation of cell adhesion plays a central role during gastrulation (reviewed in [27]). Data presented in this paper complements these findings by demonstrating that the role of cadherins extends beyond gastrulation, to orchestrate posterior body formation.

## Conclusion

Formation of the vertebrate tail involves a continuation of gastrulation-type movements that shape the head and trunk region and posterior-specific behaviors [8]. While the cadherin superfamily has a well established role in mediating mesodermal morphogenesis during gastrulation, less is known about the function of cadherins in lengthening the posterior body region. We provide here several pieces of evidence that N-cad and other members of the classical cadherin subfamily are essential for eversion of the tailbud and to a lesser extent for shaping the mesoderm during gastrulation. Consistent with these observations, zebrafish *N-cad *is expressed in axial and paraxial mesoderm during gastrulation and throughout the tailbud. Moreover, *N-cad *appears to interact synergistically with *vangl2*, a member of the non-canonical Wnt signaling pathway to mediate tailbud eversion. Together these findings further highlight the central role of members of the cadherin superfamily in the cell behaviors that shape the vertebrate embryo.

## Methods

### Zebrafish maintenance, embryo generation, staging

Zebrafish embryos (*Danio rerio*) were collected from mated adult fish within 30 minutes post fertilization and maintained at 28.5°C until the desired developmental stage was reached [87]. Offspring from: wild type, AB and TL; *N-cad *heterozygotes, *N-cad*^*p79emcf*^, *N-cad*^*m117 *^and *N-cad*^*r2.10*^*; tri*^*m747 *^heterozygotes and *kny*^*hi1688 *^heterozygotes were used.

### Morpholino injections

Antisense *N-cad *morpholino oligonucleotides (MO) were generated against the translation initiation start site of zebrafish *N-cadherin *(Gene Tools; [48]):

*N-cad *MO: 5'TCTGTATAAAGAAACCGATAGAGTT-3'

This MO targets *N-cad *efficiently, as immuno-labeling using an antibody against zebrafish N-cad did not detect any signal in embryos injected with moderate concentrations of MO (Additional file 5) and a genome-wide database search yielded no significant hits other than *N-cad*.

Antisense *vangl2 *MO (also called *stbm *MO) were generated against the translation initiation start site of zebrafish *vangl2 *as previously described (Gene Tools; [15]):

*vangl2 *MO: 5'GTACTGCGACTCGTTATCCATGTC-3'

MO stock solution (10 mg/ml) was diluted to desired concentrations in *Danio *water. Embryos were injected with *N-cad *MO (0.8 ng) or *vangl2 *MO (0.4 ng, 0.48 ng, 0.56 ng, 0.64 ng, 0.72 ng, 0.8 ng) at the 1- to 2- cell stage using a nitrogen-pressured microinjector (Harvard Apparatus). To ensure accuracy of results, MO injections were carried out using the same microinjection needle per injection series. In addition, injections for each series of experiments were done on the same day and comparisons were done between siblings whenever possible. At the appropriate stage, embryos were either imaged live or fixed for *in situ *hybridization or/and immunocytochemistry.

### *In situ *hybridization

*In situ *hybridization was performed as described in Thisse (1993; [88]). To synthesize antisense digoxigenin RNA probes, *N-cad *[89] was linearized with *Hind*III and transcribed with T7 polymerase, *myoD *[90] was linearized with *Bam*HI and transcribed with T7 polymerase, *no tail *[91] was linearized with XbaI and transcribed with T7, *dlx3 *[92] was linearized with *Sal*I and transcribed with T7 polymerase, *papc *[33] was linearized with *Apa*I and transcribed with T3 polymerase.

### Antibody and other labeling

Immunocytochemistry was carried out as described in Westerfield (2000; [93]). Primary antibodies: rabbit α-Sox3C [94], working concentration of 1:2000 dilution; rabbit α-N-cad [95], working concentration of 1:100 dilution; and α- β-catenin (BD Transduction Laboratories), working concentration of 1:200. Detection of primary antibodies was carried out using fluorescein-conjugated secondary antibodies: Cy3 α-rabbit (Biomedia), working concentration of 1:200 dilution; Alexa 488 α-rabbit, working concentration of 1:200 dilution; or/and Alexa 488 α-mouse, working concentration of 1:200 dilution. DAPI was used according to the manufacturer's instructions (Molecular Probes).

### *In situ *detection of apoptosis in whole mounts

Detection of apoptotic cells was carried out using the *In situ *Cell Death Detection Kit, Fluorescein (Roche). Labeling on whole mount embryos was carried out according to Cole and Ross (2001; [96]) excluding the color reaction. Optical sections of the tail region were taken using a confocal microscope and cells were counted in three different subregions (refer to Table 1 and Figure 6).

### Tail length measurements

Tail length measurements were made on Nomarski images of live embryos, using the OpenLab software. Measurements were taken from the beginning of the yolk extension to the tip of the tail.

### Cell labeling/movement analysis

Injection and uncaging of fluorescein in cell groups for cell movement analysis was carried out as described by Sepich et al., 2000 [97], with slight modifications. Anionic dextran DMNB caged fluorescein, 10,000 MW (Molecular Probes, D-3310) was dissolved in 120 mM KCl, 20 mM Hepes pH 7.5 to a final concentration of 1% [98]. Before use, the dye was centrifuged 3 to 5 minutes in a microfuge. A small quantity (1 nl) was injected into the yolk of 1- to 2- cell stage embryos using a nitrogen-pressured microinjector (Harvard Apparatus). Embryos at the 3–4 som stage were dechorionated, anesthetized with tricaine (Sigma), and positioned dorsal side up on a depression slide filled with 3% methycellulose (Sigma). Uncaging was performed with the 440 nm beam from a Photonic Instruments MicroPoint laser system mounted on a Zeiss Axiophot microscope and focused through a 20× objective. Embryos were imaged at 18 hpf using a Zeiss Axioskop 2 microscope.

### Imaging and sectioning

Imaging of live specimens – At the desired developmental stage embryos were dechorionated, placed in imaging solution containing 3% methylcellulose (Sigma) with MESAB (4 mg/ml ethyl-*m*-aminobenzoate methanesulphonate, 1% Na_2_HPO_4_) (1:100 dilution) and imaged using a Zeiss Axioskop 2 compound microscope.

Imaging of fixed preparations – Whole-mount embryos or sections labeled using *in situ *hybridization were mounted in 100% glycerol (whole mounts) or PBS (sections) and imaged using a Zeiss Axioskop 2 compound microscope. Fluorescently labeled specimens, (whole mounts or sections) were placed in Aqua Poly/Mount (Polysciences, Inc.) and imaged using a Zeiss LSM 510-Meta confocal microscope.

Sectioning – Embryos were sectioned using a vibratome (Vibratome, Inc.), as described in Hong and Brewster, 2006 [49].

## Authors' contributions

MH designed and interpreted the experiments with RB and contributed to the draft of the manuscript. MH also carried out: morpholino injections, determining the optimal concentrations to use for these studies; the *N-cad *mRNA expression analysis; the immunolabeling using α-Sox3C and α-N-cad; the cell movement analysis using caged fluorescein; the *in situ *cell death detection assay; imaging of live embryos.

EH helped to design experiments and draft the manuscript. EH also performed: the expression analysis of *ntl, dlx3*, and *papc *in WT and mutant embryos and the immunolabeling analysis of N-cad expression.

OF assisted with morpholino injections, contributing to the initial observation that *N-cad *and *vangl2 *interact.

RB designed and interpreted experiments in addition to coordinating and supervising the research efforts. RB obtained the funding to support for this research project.

All authors read and approved the final manuscript.

## Supplementary Material

Additional file 1 **Loss of *N-cadherin *causes posterior neural tube defects**. Cross sections through the posterior domain of the yolk sac extension of 30 hpf WT **(A) **and *N-cad *morpholino-injected (0.8 ng) **(B) **embryos labeled with α-Sox3C (pink) and DAPI (blue). Dotted line delineates the shape of the NT. Scale bar, 20 μm. Click here for file

Additional file 2 ***N-cad*^*m117 *^heterozygotes embryos have mild gastrulation defects**. Width of *papc *domain in *N-cad*^*m117*^mutants is comparable to WT embryos. Measurements of *papc *expression domain in WT, offspring from a cross between WT and *N-cad*^*m117/+ *^heterozygous fish, and *N-cad*^*m117 *^homozygous mutant embryos. Click here for file

Additional file 3 **Titration of *vangl2 *MO in WT embryos**. Injection of *vangl2 *MO into WT embryos results in shortening of tail length. Tail lengths of wildtype uninjected and wildtype injected with *vangl2 *MO (0.4 ng, 0.48 ng, 0.56 ng, 0.64 ng, 0.72 ng, and 0.8 ng) embryos were measured at 30 hpf. Click here for file

Additional file 4 ***vangl2 *and *kny *do not regulate N-cad expression or localization**. Cross-sections through the tail region of 18 som embryos labeled with α-N-cad (green) and DAPI (blue). N-cad is localized at the plasma membrane in WT **(A)**,*tri ***(B) **and *kny *mutant **(C) **embryos. Insets show a higher magnification of N-cad labeling in the mesoderm. Dotted white circles show the location of the notochord. Scale bar, 20 μm. Click here for file

Additional file 5 ***N-cad *MO prevents translation of N-cad protein**. Cross-sections through the tail region of 30 hpf embryos labeled with α-N-cad (green) and DAPI (blue). In WT, uninjected embryos **(A) **N-cad protein is expressed throughout the neural tube, where it is enriched at the apical surface (red arrowhead). In addition, N-cad is observed in the notochord and postmigratory slow cells (PSCs). Labeling is absent in the N-cad morpholino-injected (0.8 ng) **(B) **embryos. Abbreviations: NT, neural tube; som, somite; NC, notochord. Scale bar, 10 μm. Click here for file
