# Supplementary figures and images for: Cadherin-mediated adhesion regulates posterior body formation
Source: BMC Dev Biol. 2007 Nov 28;7:130. doi: 10.1186/1471-213X-7-130 (PMC2231375; doi:10.1186/1471-213X-7-130)

**A, WT**

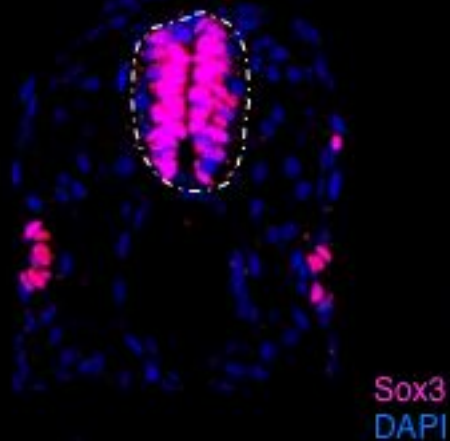

**B, *N-cad* MO**

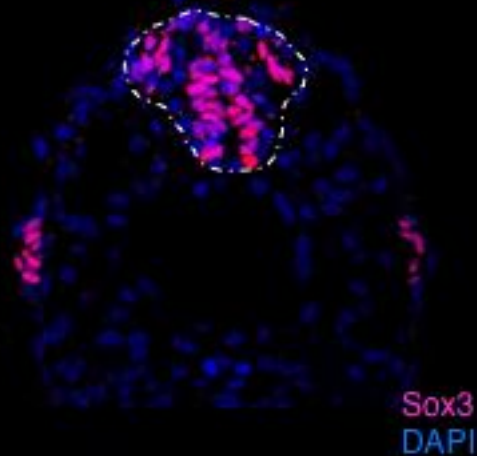

Supplement: Additional file 1 — Loss of N-cadherin causes posterior neural tube defects. Cross sections through the posterior domain of the yolk sac extension of 30 hpf WT (A) and N-cad morpholino-injected (0.8 ng) (B) embryos labeled with α-Sox3C (pink) and DAPI (blue). Dotted line delineates the shape of the NT. Scale bar, 20 μm. [file 1471-213X-7-130-S1.pdf]

# ***N-cad*<sup>m117</sup> heterozygote embryos have mild gastrulation defects**

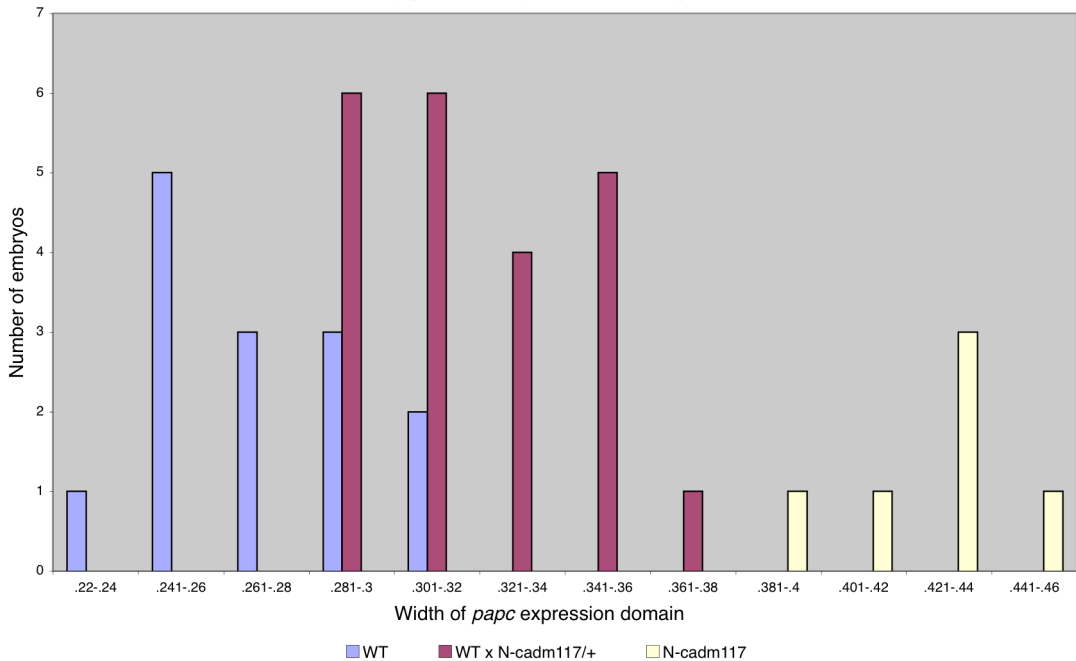

Supplement: Additional file 2 — N-cadm117 heterozygotes embryos have mild gastrulation defects. Width of papc domain in N-cadm117mutants is comparable to WT embryos. Measurements of papc expression domain in WT, offspring from a cross between WT and N-cadm117/+ heterozygous fish, and N-cadm117 homozygous mutant embryos. [file 1471-213X-7-130-S2.pdf]

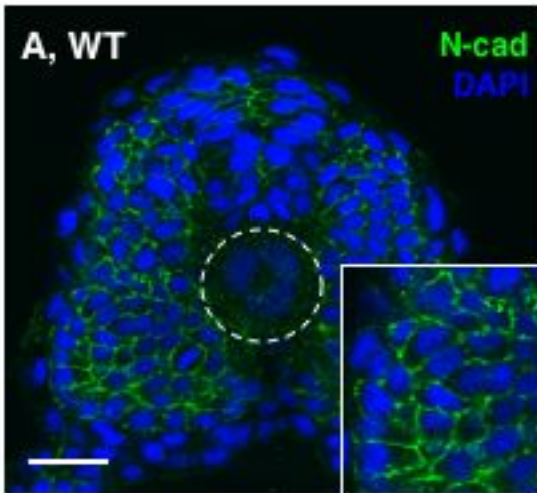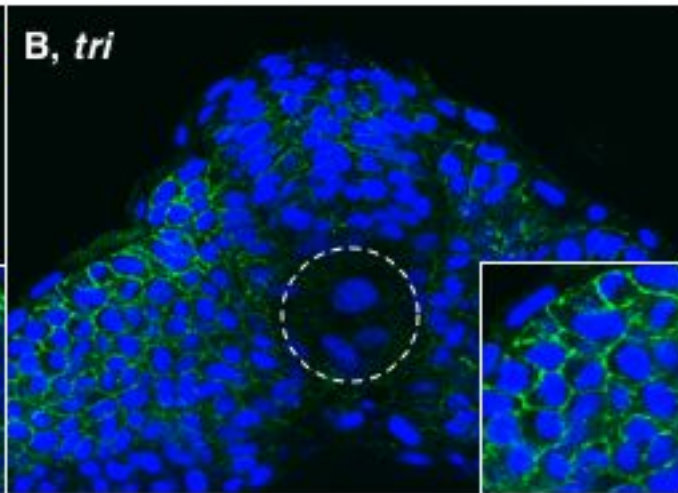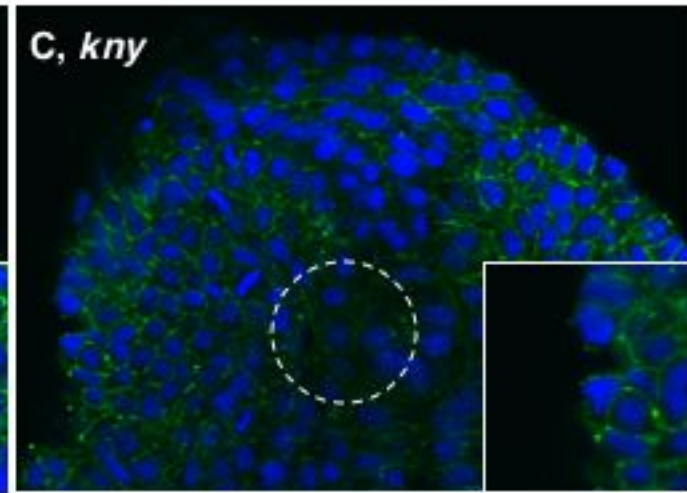

Supplement: Additional file 4 — vangl2 and kny do not regulate N-cad expression or localization. Cross-sections through the tail region of 18 som embryos labeled with α-N-cad (green) and DAPI (blue). N-cad is localized at the plasma membrane in WT (A),tri (B) and kny mutant (C) embryos. Insets show a higher magnification of N-cad labeling in the mesoderm. Dotted white circles show the location of the notochord. Scale bar, 20 μm. [file 1471-213X-7-130-S4.pdf]

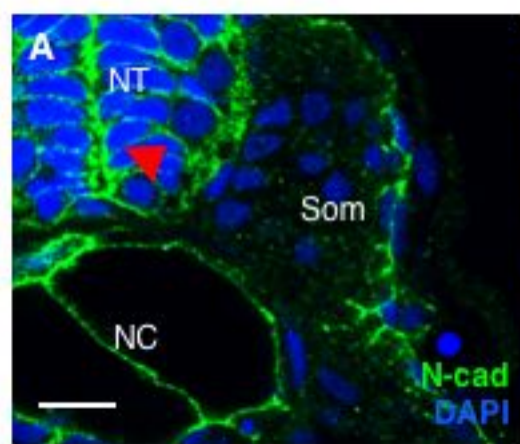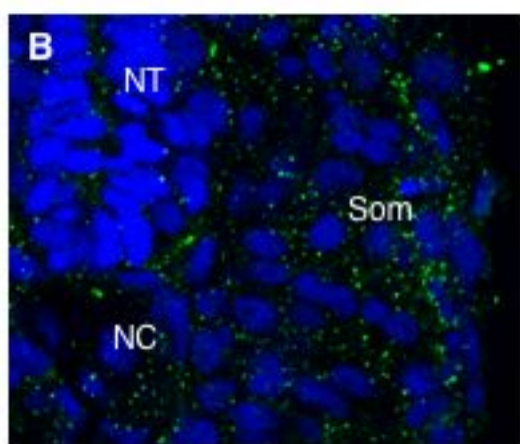

Supplement: Additional file 5 — N-cad MO prevents translation of N-cad protein. Cross-sections through the tail region of 30 hpf embryos labeled with α-N-cad (green) and DAPI (blue). In WT, uninjected embryos (A) N-cad protein is expressed throughout the neural tube, where it is enriched at the apical surface (red arrowhead). In addition, N-cad is observed in the notochord and postmigratory slow cells (PSCs). Labeling is absent in the N-cad morpholino-injected (0.8 ng) (B) embryos. Abbreviations: NT, neural tube; som, somite; NC, notochord. Scale bar, 10 μm. [file 1471-213X-7-130-S5.pdf]
