# Supplementary material for: Cadherin-mediated adhesion regulates posterior body formation
Source: BMC Dev Biol. 2007 Nov 28;7:130. doi: 10.1186/1471-213X-7-130 (PMC2231375; doi:10.1186/1471-213X-7-130)
Supplement: Additional file 3 — Titration of vangl2 MO in WT embryos. Injection of vangl2 MO into WT embryos results in shortening of tail length. Tail lengths of wildtype uninjected and wildtype injected with vangl2 MO (0.4 ng, 0.48 ng, 0.56 ng, 0.64 ng, 0.72 ng, and 0.8 ng) embryos were measured at 30 hpf. [file 1471-213X-7-130-S3.doc]

**Additional file 3. Injection of *vangl2* MO into WT embryos results in shortening of tail length.** Tail lengths of wildtype uninjected and wildtype injected with *vangl2* MO (0.4ng, 0.48ng, 0.56ng, 0.64ng, 0.72ng, and 0.8ng) embryos were measured at 30 hpf.
